# Relapse and frequency of injection of monthly paliperidone palmitate—A retrospective case–control study

**DOI:** 10.1192/j.eurpsy.2021.4

**Published:** 2021-02-03

**Authors:** Emily Laing, David Taylor

**Affiliations:** 1 Pharmacy, Cwm Taf Morgannwg University Health Board, Abercynon, Rhondda Cynon Taff CF45 4SN, United Kingdom; 2 Pharmacy Department, Maudsley Hospital, London SE5 8AZ, United Kingdom

**Keywords:** Antipsychotic, depot, paliperidone, relapse

## Abstract

**Background:**

Outcomes with long-acting injections (LAIs) are generally better than with oral antipsychotic therapy. However, the use of LAIs does not assure compliance with treatment and in clinical practice patients often miss injections or receive their injections later than intended.

**Method:**

We conducted a case–control study to identify demographic and treatment associations with relapse (cases) on paliperidone 1-monthly injection (PP1M) compared with age-, gender-, and ethnicity-matched controls.

**Results:**

We identified 16 cases and matched 43 controls. Baseline variables did not differ except that cases had received significantly more antipsychotic drugs before initiation with PP1M (3.94 vs. 2.12; *p* < 0.001). Cases had fewer PP1M injections administered compared with the control group (9.69 vs. 11.37; *p* < 0.001) and this group had a longer interval between injections than the control group (37 vs. 33 days; *p* < 0.001).

**Conclusions:**

Relapse on PP1M is associated with reduced frequency of injection and a longer interval between doses.

## Introduction

In schizophrenia, there are strong links between poor adherence with oral antipsychotic therapy and relapse, admission, and increased NHS costs [1]. It is assumed that the lower adherence to prescribed medication, the poorer the outcome. Certainly, after complete cessation of an antipsychotic medication, relapse is almost inevitable [2].

The use of long-acting injections (LAI) formulations significantly reduces relapse compared with oral treatment [3]. Most mirror image studies show a clinically significant reduction in hospital admissions and bed days in the period after paliperidone LAI was initiated [4, 5]. However, this reduction in hospital use seems to be lost if injection frequency falls below a certain threshold [6].

The aim of this study was to assess the association between relapse while prescribed PP1M and frequency of injection.

## Method

This was a retrospective, observational case control study that was undertaken in the South London and Maudsley NHS Foundation Trust in the United Kingdom. All patients prescribed monthly paliperidone palmitate (PP1M) LAI for longer than 12 months between January 2016 and January 2020 were included. Patients on 3-monthly paliperidone (PP3M) were excluded. All patients had an F20 schizophrenia diagnosis (i.e., any F20xx diagnostic code listed in their record).

Patients were identified using the trust’s PP1M database which captures all patients starting PP1M in normal clinical practice (fully described elsewhere [5, 7]). Patients were separated into case or control groups. Case inclusion was all patients who had had a relapse of illness defined as admission to a mental health hospital or transfer to Home Treatment Team (HTT) from a lower level of care (i.e., from specialist psychiatric care only as an out-patient or care received only from a general practitioner). Controls were then matched to the cases using age (±5 years), ethnicity, gender, and PP1M prescribed for at least 12 months without relapse. Where control patients were prescribed PP1M for longer than 12 months, the previous 12 months before the date of data collection was used to calculate number of injections received and interval between injections.

Demographic details were taken from the database and clinical information was obtained using patients’ electronic medical notes. Smoking status was obtained from electronic notes and was determined as any mention of tobacco smoking during the 12 months of the analysis period. History of alcohol or substance misuse was also gleaned from electronic notes; this misuse could have taken place at any time before the end of the analysis period.

Baseline demographic data for cases and controls are included in Table 1. For cases, the number of PP1M injections administered over the 12 months preceding the relapse date were counted. The two initial loading doses were counted as only one injection for ease of analysis. The interval between each PP1M injection or, if missed, the interval between the last PP1M injection and admission date were recorded for the same 12-month period. A re-loading dose regimen is required if an injection is more than 2 weeks late. We recorded when re-loading was required, why it was required, and when it was administered for cases and controls.

**Figure 1.**
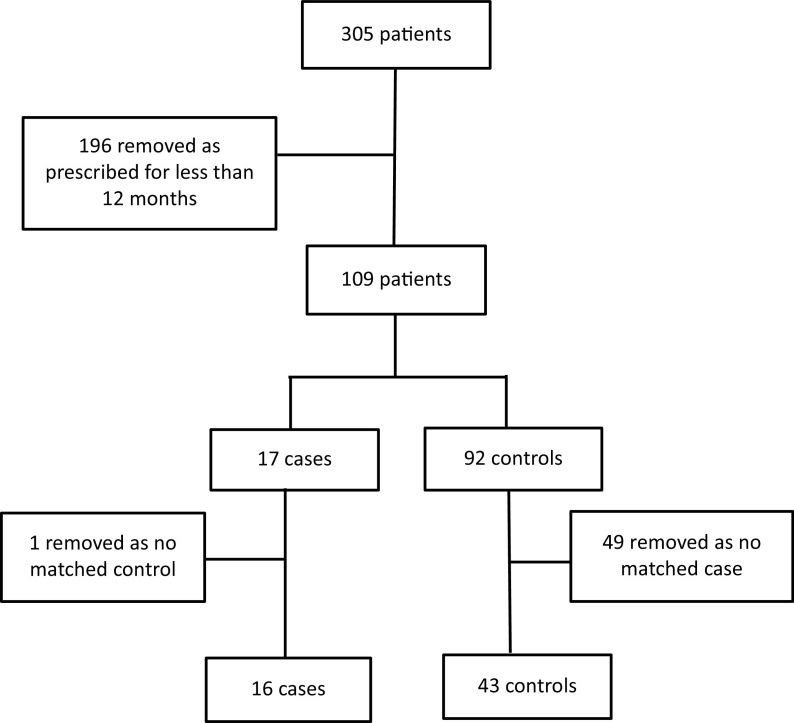
Schematic representation of method.

**Table 1.** Demographic data.

Parameters	Cases *N* = 16	Controls *N* = 43	*p* value
Mean age at initiation (years)	41.38	44.26	0.45
Male gender [*n* (%)]	16 (100)	43 (100)	
Mean years of psychiatric illness	12.69	12.16	0.77
Ethnicity [*n* (%)]			
Black	11 (69)	32 (74)	0.66
White	5 (31)	11 (26)	
Mean number of antipsychotics tried pre PP1M	3.94	2.12	<0.001
Clozapine Previously tried [*n* (%)]	1 (6)	0 (0)	
Mean PP1M dose (mg/month)	112.5	125	0.53
Smoking status			
Y [*n* (%)]	10 (62.5)	30 (70)	0.60
N [*n* (%)]	6 (37.5)	13 (30)	
Substance misuse history			
Y [*n* (%)]	10 (62.5)	31 (72)	0.48
N [*n* (%)]	6 (37.5)	12 (28)	
Alcohol misuse history			
Y [*n* (%)]	8 (50)	19 (44)	0.69
N [*n* (%)]	8 (50)	24 (56)	

Compliance was defined as 80% of prescribed LAIs administered over the 12-month period, that is 10 or more injections. All cases were followed-up to see what antipsychotics were prescribed immediately after relapse.

### Statistical analysis

All statistical tests were performed using Microsoft Excel 2013. Descriptive statistics were used to summarize patient and medication baseline data. Means and standard deviations (SDs) were calculated for continuous data with paired Student’s *t* test performed to compare cases and controls. Categorical data were compared using Chi-squared test. Statistical significance was considered demonstrated if *p* value was less than 0.05.

## Results

The PP1M database had 305 patients recorded. Patients prescribed PP1M for less than 12 months were removed (196 patients) which left 17 potential cases (relapse patients) and 92 potential controls. One of the 17 potential cases was excluded as no matched controls were found. In total, 49 controls were excluded as they could not be matched leaving 16 cases and 43 controls. The one excluded case was a female. There were 22 female controls, but these did not match the case so were excluded. All participants in the study were male.

Demographic information for the cases and control groups are given in Table 1. Of the 16 relapses, 14 were admitted to a mental health hospital and 2 were transferred from a lower level of care to HTT. For the cases, the mean number of months that a patient was prescribed PP1M before relapse was 22.5 with a range of 12–37 months. Table 1 also shows baseline data. The number of antipsychotics trialed before PP1M was the only baseline parameter that showed a statistically significant difference between cases and controls (*p* < 0.001).

Cases had statistically significantly fewer injections administered compared with the control group and the case group had a statistically significant longer interval between injections than the control group (Table 2).

**Table 2.** Frequency and interval time between LAI administration comparison table.

Parameters	Cases *N* = 16	Controls *N* = 43	*p* value
Mean number of injections administered over 12 months	9.69	11.37	0.0009
Mean interval between injection and/or relapse (days)	37.24	32.51	0.0005

Of the 16 cases, 9 (56%) were defined as compliant, whereas, 39 (91%) of the 43 controls were compliant (Table 3). Two patients in each group were administered 13 injections within the 12 months. All other patients received 12 or fewer injections.

**Table 3.** Compliance associated with relapse comparison table.

Parameters	Cases *N* = 16	Controls *N* = 43	*p* value
Compliant [*n* (%)]	9 (56)	39 (91)	0.0025
Noncompliant [*n* (%)]	7 (44)	4 (9)	

After relapse, 9 of 16 (56%) of the cases continued on a formulation of paliperidone LAI (monthly or 3-monthly). Of those remaining, four were switched to clozapine, one was switched to aripiprazole, one did not have any antipsychotic restarted, and for one documentation was unclear.

In total, 11 patients were considered noncompliant. Of the four controls in this group, three were maintained on PP1M and one switched to PP3M. Of the seven cases, four were maintained on PP1M, one was switched to aripiprazole, one documentation was unclear, and one did not have any antipsychotic restarted.

Re-loading regimens were clinically indicated on 14 occasions in the 16-patient case group with 3 (21%) being administered, and on 17 occasions in the 43-patient control group, with 6 (35%) being administered (*p* = 0.001, for number of reloads indicated). Details of why an LAI was administered late (leading to reloading being indicated in 31 cases in total) was poorly documented but 7 (2 cases and 5 controls) cited patient disengagement, and 6 (3 cases and 3 controls) cited patient refusal to accept an injection.

## Discussion

This study demonstrated an association between relapse and a lower number of PP1M injections administered over a 12-month period. This is in accordance with previous work. A study in 2008, explored adherence in 38 patients receiving oral and 12 patients on LAI treatment. The LAI treatment group were divided into either adherent or nonadherent. A reduction in relapse and hospitalization was seen in only the adherent group [8]. More recently, a 6-year mirror-image study with 173 participants was published. It was concluded that there was a direct association between partial compliance and re-hospitalization in patients prescribed PP1M. It was also found that fully compliant patients (100% of prescribed injections administered) maintained the best outcomes in terms of reduced bed days [6].

There are a number of limitations to this study, such as all participants being male and all having a F20.xx diagnosis, and so results may not be replicable in other cohorts. Another limitation is the potential for inaccuracy in documentation in the electronic records. Perhaps most importantly, relapse appeared to be associated with prior use of a greater number of antipsychotics. This clearly confounds the observation that caseness was associated with fewer injections given and may offer a partial explanation for increased risk of relapse.

In total, four of the case group were switched to clozapine after the relapse, all of whom were within the compliant group of cases. This seems an appropriate next step for this subgroup who failed on appropriate doses of PP1M.

Many clinicians imagine that using a LAI for treatment will solve the problem of poor adherence. In reality, patients are at liberty to exercise their right not to attend for a scheduled injection or to refuse to receive the injection. So, the use of an LAI improves the clinician’s awareness of poor/noncompliance, but may do little to improve actual compliance. Our results confirm that relapse is more likely when compliance is poor even with an LAI formulation. This research will hopefully lead to a more considered approach when making a decision around the best formulation for a patient, including patient engagement in treatment options to attempt to improve adherence. There are still significant benefits with LAIs compared to oral antipsychotic medication due to reduced relapse rates and the clarity of treatment compliance to aid clinical decision making.

## Data Availability

The data that support the findings of this study are available from the corresponding author, D.T., upon reasonable request.
